# Quantitative morphological changes in the interplacentomal wall of the gravid uterine horn of cattle during pregnancy

**DOI:** 10.1186/s12958-015-0030-3

**Published:** 2015-04-18

**Authors:** Rose M Albers, Anke Schnapper, Martin Beyerbach, Alois Boos

**Affiliations:** Institute of Veterinary Anatomy, University of Zurich, Vetsuisse Faculty, Winterthurerstrasse 260, 8057 Zurich, Switzerland; Institute of Functional and Applied Anatomy, Hannover Medical School, Hannover, Germany; Institute for Anatomy, University of Veterinary Medicine Hannover Foundation, Hannover, Germany; Institute for Biometry, Epidemiology and Information Processing, University of Veterinary Medicine Hannover Foundation, Hannover, Germany

**Keywords:** Morphometry, Stereology, Uterus, Cattle, Pregnancy

## Abstract

**Background:**

The interplacentomal wall of the gravid uterine horn in cattle is the subject of reports dealing mainly with specific aspects of early pregnancy or the peripartal period. Only a very limited number of early and descriptive studies includes the whole period of pregnancy. Thus, there is a gap concerning quantitative morphological data of the uterine wall during pregnancy. We hypothesized that the specific requirements of pregnancy are reflected by significant and characteristic morphologic changes.

**Methods:**

Interplacentomal segments of the fetus-bearing horn of the uterus of 47 cows were collected at slaughter, assessed quantitatively by light microscopy, grouped into trimesters (trim), and data were analyzed statistically.

**Results:**

During pregnancy there were significant increases (p<0.05) in the measured parameters: heights of the endometrial surface epithelium (31 increased to 46 and 46 μm, in the 1st, 2nd and 3rd trim, respectively), glandular epithelium (19.6 to 22.4 and 25.4 μm, respectively), diameters of glands (94 to 166 to 239 μm, respectively) and glandular lumina (56 to 122 to 188 μm, respectively). Volume density of the glandular epithelium did not change, while that of glandular lumina increased significantly (8 to 26 to 40% in the 1st, 2nd and 3rd trim, respectively) and of endometrial stroma decreased with ongoing pregnancy (67 to 46 to 37%; p<0.05).

Diameters of myometrial smooth muscle cells (MSMC) (9.7 to 12.4 and 12.9 μm, respectively, for the 1st, 2nd and 3rd trim; p<0.05), and the volume fraction of myometrial stroma increased (6 to 10 to 13%; p<0.05), while decreases were observed in MSMC nuclear volume density (4.4 and 4.0 to 2.4%; p<0.05). The fraction of MSMC cytoplasm (89 to 85%) and the nucleus:cytoplasm ratio (0.05 to 0.03%) both decreased for the 1st *vs.* 3rd trim, respectively (p<0.05).

**Conclusions:**

These results indicate that the interplacentomal wall of the gravid uterine horn is subjected to significant morphological changes during pregnancy, underlining the importance of endometrial surface epithelium and of gland hypertrophy for nourishment of the conceptus, of increased myometrial extracellular matrix for uterine tensile strength and of myometrial smooth muscle hypertrophy for expulsion of the fetus at term.

## Background

The interplacentomal wall of the gravid uterine horn in cattle has, besides many other important functions, three major tasks during pregnancy: secretion of uterine milk, provision of uterine tensile strength and mechanical stability, and expulsion of the fetus at term.

To meet these requirements, the uterus is supplied with endometrial glands (EG), which are essential, at least during early pregnancy, for nourishment of the growing fetus [1-3]. Fetal growth during pregnancy requires concomitant uterine enlargement and increasing tensile strength, which are paralleled by an increase in uterine mass [4-6]. In placentomes, the latter is accomplished predominantly by hyperplasia [6-10]. Within the interplacentomal uterine wall, however, hypertrophy is the main contributor to tissue gain, as revealed by qualitative, *i.e.*, non-quantitative morphological, biochemical and histochemical studies [6,7,10-15]. Synthesis of interstitial matrix proteins at specific sites [16,17] and accumulation of contractile proteins [18-21] augment uterine wall tensile strength and contractile force. We therefore hypothesized that the specific requirements of pregnancy are reflected by significant and characteristic morphological changes and that the cause of the increased interplacentomal uterine mass, *i.e.,* hypertrophy, is reflected by low mitotic indices.

Although some early measurements on uterine glands are available, made almost 50 years ago, they lack adequate statistical analysis [13] and no recent quantitative and thoroughly statistically evaluated data encompassing all structural elements of the uterine wall are available on the morphological changes occurring within the interplacentomal wall of the gravid uterine horn of cattle. In the present study, appropriate specimens were analysed morphometrically and also, for the first time, stereologically. The results are discussed in the light of histochemical findings obtained from the same samples [15,16,21] and from other reports published elsewhere using qualitative morphological and functional parameters [for references see below].

## Methods

The uteri of pregnant Holstein–Friesian cows were collected at an abattoir (Vosding, Gleidingen, Germany) and 5 to 6 organs for each of the months 1–9 of gestation were selected at random for the present study. Within 30 min after the animals were killed, they were eviscerated, the pregnant uteri opened and the crown-rump length of the 47 embryos or fetuses present was recorded to estimate fetal age, as follows: up to 1.5 cm, 1 month (n=4); 2–4 cm, 2 months (n=5); 6–8 cm, 3 months (n=5); 15–23 cm, 4 months (n=6); 26–32 cm, 5 months (n=6); 36–44 cm, 6 months (n=5); 48–55 cm, 7 months (n=6); 60–67 cm, 8 months (n=5); and 70–82 cm, 9 months (n=5) [22]. In one case out of the four first month specimens, the tiny embryo was not found macroscopically, thus, the uterine contents of the early pregnant animal were examined histologically so that the embryonic tissues could be detected.

### Tissue sampling and processing

A piece of the interplacentomal wall of the gravid uterine horn measuring 3 cm × 3 cm and the adherent allantochorion were excised from each animal. The tissues were cut into three strips of equal width and immersed in 4% neutral buffered formaldehyde solution (v/v) according to Lillie [23] for 24 h, rinsed in tap water, dehydrated in a graded series of ethanol and acetic acid-n-butyl ester (Riedel-de-Haën, Seelze, Germany) and embedded in Paraplast Plus(R) (Sherwood Medical, St. Louis, MO, USA).

### Histological techniques and staining procedures

Tissues were cut at 5 μm (1140/Auto-cut(R), Reichert-Jung, Heidelberg, Germany), then sections were mounted onto glass slides and routinely stained with hematoxylin-eosin and Masson-Goldners trichrome stain [23].

### Measurements

All measurements were performed at a magnification of x400 using a computerized and calibrated measuring device (CUE 3(R), version 4.5, Galai Productions Ltd., Migdal Haemek, Israel). Slides, one per animal, were assessed on a video screen.

The following parameters were evaluated using point-to-point distance measurement with the computer mouse: (I) height of the endometrial surface epithelium (SE), (II) height of the epithelium of intermediate and deep endometrial glands (IGE, DGE, respectively), (III) diameter of intermediate and deep glands (IG, DG), respectively, (IV) diameter of the lumen of IG and DG, and (V) diameter of myometrial smooth muscle cells (MSMC). Epithelial heights were measured at 50 randomly selected locations for each slide. Gland and gland lumen diameters were assessed at 30 locations in randomly selected round profiles. MSMC diameter was measured in 50 round profiles per section, *i.e.*, per animal.

To analyze the differential structural and cellular compositions of the interplacentomal uterine wall stereologically, volume densities (Vv) were estimated. For this, a transparent test lattice of point probes, each represented by the crossing of equidistant vertical and horizontal lines and representing 500 cross-points, was used. Cross-points situated exactly on the border between two adjacent structures accounted for the structure extending to the left and downwards from the cross-point. Three randomly selected endometrial and myometrial tissue fields, each representing 500 cross-points, were assessed for the following elements: (I) endometrial stroma, glandular epithelium, glandular lumen, and (II) myometrial stroma, MSMC cytoplasm and MSMC nuclei. These data were used to calculate Vv of these elements within the endometrium and myometrium, respectively. Finally, the nucleus:cytoplasm ratio (NCR) was assessed for SE and IGE/DGE by counting nuclear and cytoplasmic hits within the epithelia, each performed in 10 randomly selected microscopic fields and encompassing at least 500 cross-points as well as the NCR of MSMC.

### Statistical methods

Mean values of morphometric and stereologic data of a section, *i.e.,* representing a single animal, were used for further analysis. Animals were grouped into trimesters (trim 1–3, *i.e.*, months 1–3 (n=14), months 4–6 (n=17) and months 7–9 (n=16), respectively). Data were analyzed statistically using SAS(R) software (SAS Institute Inc., Gary, NC, USA). After checking for normal distribution, analysis of variance (ANOVA) and the Tukey test for *post hoc* analysis were used to evaluate the influence of time (*i.e.,* trim). The Tukey test uses the experiment-wise error rate. Differences with a P-value < 0.05 were designated as significant.

## Results

### Histomorphology

**Figure 1:**
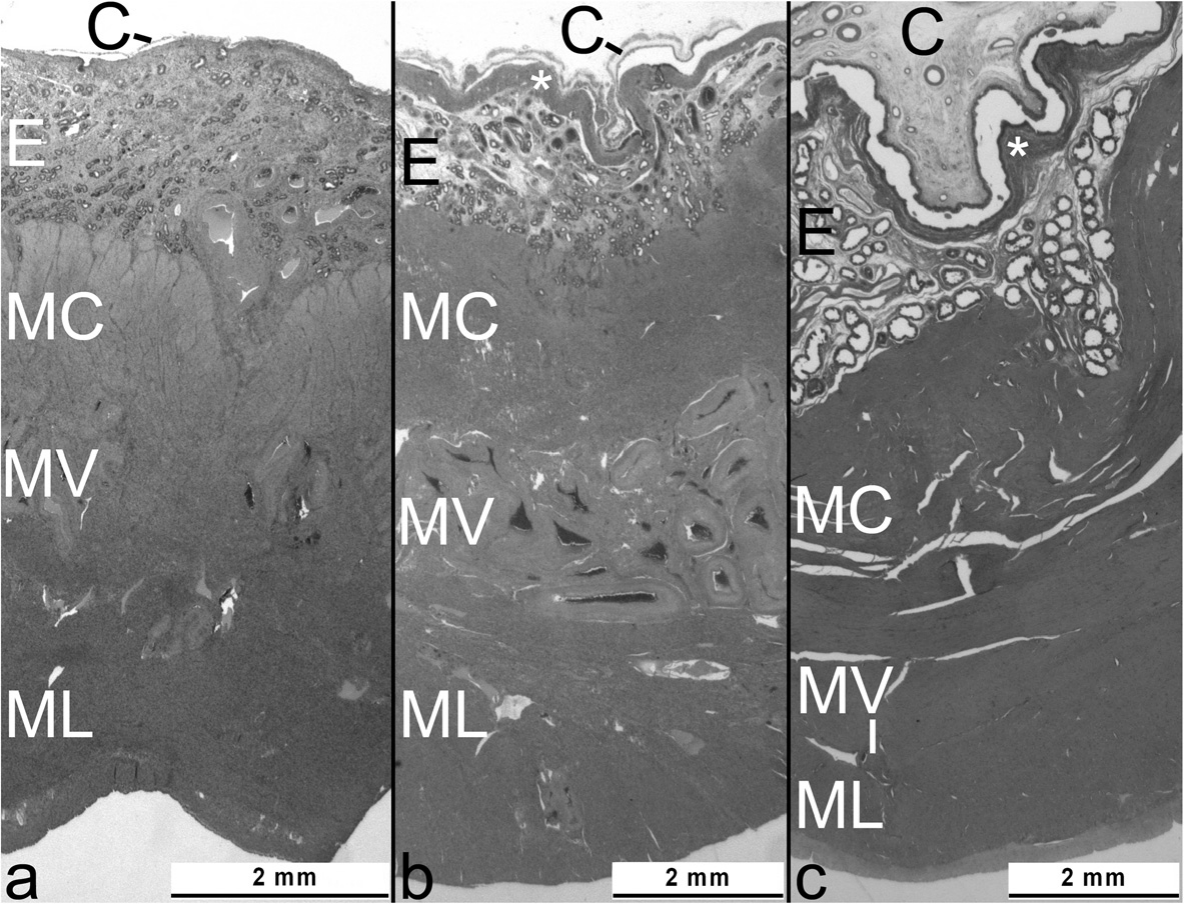
Transverse section through the uterine wall of the pregnant horn and adherent chorion. Specimens were collected at the end of gestation months 1 **(a)**, 3 **(b)**, and 8 **(c)**. Note the increasing thickness of the chorion (C) and the subepithelial connective tissue sheet (*), and the increasing diameter of deep uterine glands within the endometrium (E). MC: myometrium, circular layer; MV: myometrium, vascular layer and ML: myometrium, longitudinal layer.

**Figure 2:**
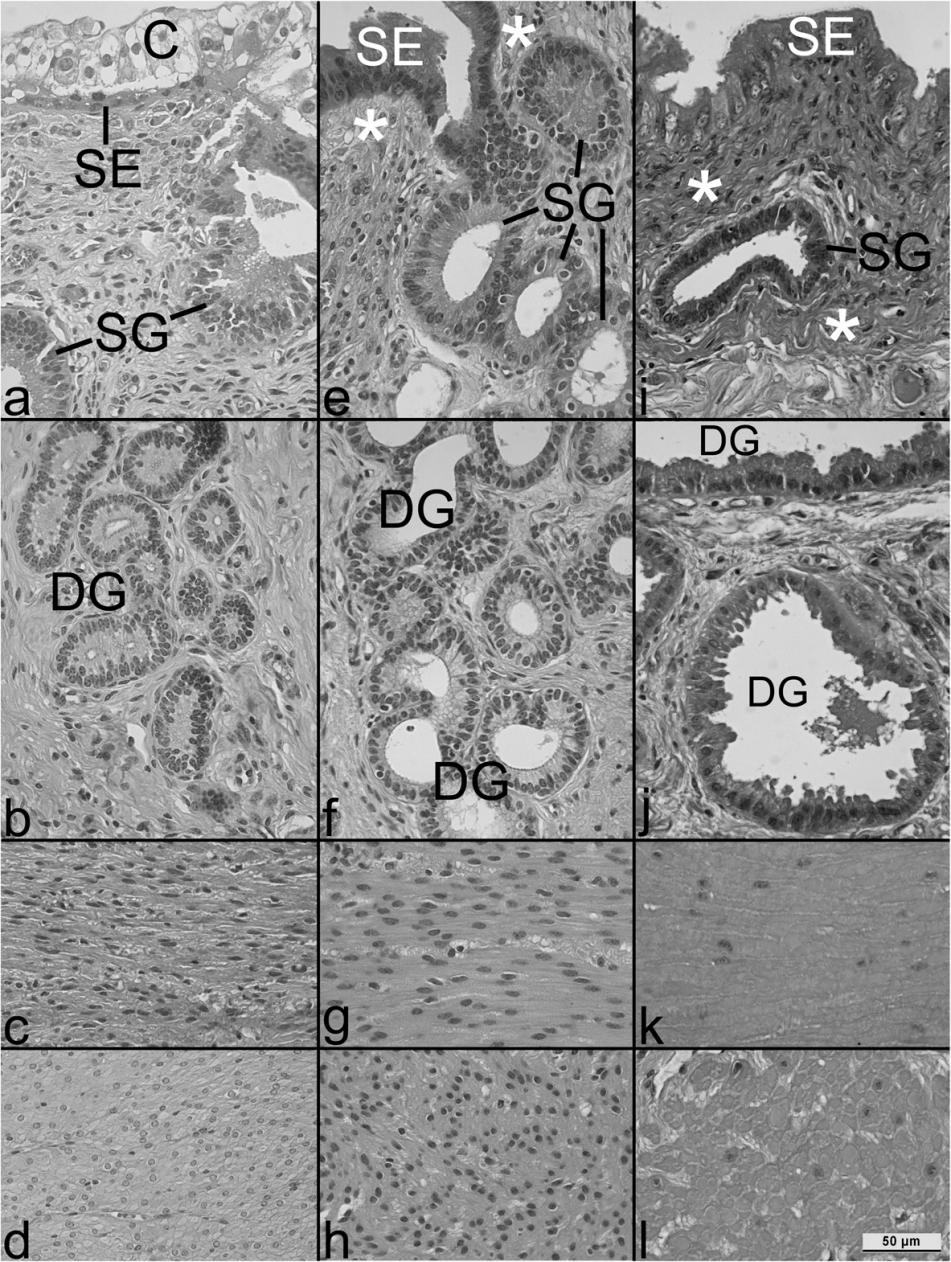
Morphology of the structural elements of the uterine wall at a higher magnification. Stratum compactum **(a,e,i)** and spongiosum endometrii **(b,f,j)**, circular **(c,g,k)** and longitudinal **(d,h,l)** layers of the myometrium at the end of gestation months 1 **(a-d)**, 3 **(e-h)**, and 8 **(i-l)**. Note the increasing height of the surface epithelium (SE), the increasing thickness of the subepithelial mesh of connective tissue fibres (*), the increasing diameter of deep uterine glands (DG) and myometrial smooth muscle cells, while superficial uterine glands SG did not show significant changes with ongoing pregnancy. The decreasing nucleus:cytoplasm ratio of myometrial smooth muscle cells is evident when comparing g,h *vs.* k,l. C: chorion.

The morphological differences between the interplacentomal wall segments of the pregnant uterine horns of early and late pregnant cows are shown in Figures 1 and 2. Notable were major changes in SE height, EG and EG lumen diameters, high secretory activities of EG even during late pregnancy and an increase in MSMC size, *i.e.*, length and diameter. Furthermore, a mesh of connective tissue fibers was formed immediately below the endometrial SE. Mitoses could mainly be detected in SE, but were very rare. Along the surface of the entire sections, up to 2–3 cm in length, the total number of mitoses in SE was usually below 10. More mitotic figures (24–50) could be counted in 5 animals and in 6 animals, no mitoses at all were found in SE. Four of these six specimens were obtained from cows during the 8^th^ (n=1) and 9^th^ months (n=3). Very few mitoses were visible in EG. All EG profiles of all the cows were examined and in 31 animals no mitoses could be found at all and one to five mitotic figures were visible in all EG of 17 animals. Only a very few mitotic figures could occasionally be seen in endometrial stromal cells and thus were not systematically counted or pursued. MSMC exhibited no mitoses at all.

### Morphometry and stereology

#### Endometrium

SE height was significantly lower in the 1^st^ compared to the 2^nd^ and 3^rd^ trim (Table 1). The same pattern was visible for SE NCR but no significant changes were found. Glandular openings, *i.e.*, superficial uterine glands, did not exhibit major changes during the first half of pregnancy, when data of pregnancy were considered individually. During the second half of gestation they could only be found sporadically within the sections and therefore were not analysed quantitatively. Because IG and DG did not exhibit distinct differences at the light microscopic level, data were combined (see Table 1). The height of IGE/DGE increased significantly between the 1^st^, 2^nd^ and 3^rd^ trim. NCR of IGE/DGE was lower in the 2^nd^ trim compared to the 1^st^ and 3^rd^ trim. Diameters of IG/DG and of the corresponding lumina increased significantly from the 1^st^ to the 2^nd^ and 3^rd^ trim. Vv of EG epithelium did not change during pregnancy. EG luminal Vv, however, increased significantly and continuously with ongoing pregnancy. In contrast, endometrial stroma exhibited a significant decrease in Vv with ongoing pregnancy (Table 1).

**Table 1:** **Morphometric and stereologic data of the interplacentomal uterine wall in cattle during pregnancy (mean value ± SD)**

**parameters**	**1st trimester (n=14)**	**2nd trimester (n=17)**	**3rd trimester (n=16)**
height of SE (μm)	31.26 +/− 11.87a	46.31 +/− 8.40b	45.61 +/− 5.63b
NCR of SE	0.42 +/− 0.08a	0.51 +/− 0.15a	0.51 +/− 0.09a
height of IGE/DGE (μm)	19.59 +/− 3.41a	22.42 +/− 2.02b	25.43 +/− 3.09c
NCR of IGE/DGE	0.58 +/− 0.11a	0.44 +/− 0.07b	0.54 +/− 0.12a
diameter of IG/DG (μm)	94.2 +/− 19.3a	166.0 +/− 36.6b	239.1 +/− 46.1c
lumen diameter of IG/DG (μm)	55.9 +/− 16.1a	122.4 +/− 35.8b	187.9 +/− 41.1c
Vv of EGE/EM (%)	24.6 +/− 5.5a	24.2 +/− 4.7a	22.9 +/− 5.5a
Vv of EGL/EM (%)	8.2 +/− 5.7a	26.1 +/− 10.8b	40.1 +/− 10.9c
Vv of ES/EM (%)	67.2 +/− 8.5a	46.1 +/− 6.7b	37.0 +/− 10.0c
diameter of MSMC (μm)	9.71 +/− 1.16a	12.43 +/− 1.37b	12.93 +/− 1.37b
NCR of MSMC	0.050 +/− 0.024a	0.047 +/− 0.017a	0.028 +/− 0.010b
Vv of MSMC nuclei/MM (%)	4.41 +/− 2.02a	4.00 +/− 1.41a	2.36 +/− 0.85b
Vv of MSMC cytoplasm/MM (%)	89.34 +/− 3.90a	86.34 +/− 3.29ab	85.00 +/− 5.01b
Vv of MS/MM (%)	6.11 +/− 3.03a	9.66 +/− 2.99ab	12.65 +/− 5.25b

Means with different letters are significantly different. n: number of animals; SE: endometrial surface epithelium; NCR: nucleus:cytoplasm ratio; IGE/DGE: epithelium of intermediate and deep glands; IG/DG: intermediate and deep glands; Vv: volume density; EGE: endometrial gland epithelium; EM: endometrium; EGL: endometrial gland lumina; ES: endometrial stroma; MSMC: myometrial smooth muscle cells; MM: myometrium; MS: myometrial stroma.

#### Myometrium

The diameter of MSMC (Table 1) was significantly lower in the 1^st^ trim compared to the 2^nd^ and 3^rd^ trim. MSMC NCR decreased significantly between the 2^nd^ and 3^rd^ trim. Vv of MSMC cytoplasm and nuclei decreased significantly during pregnancy, while Vv of myometrial stroma increased significantly towards term.

## Discussion

This morphometric study, for the first time also using stereological methods and an appropriate statistical analysis of the data obtained, clearly indicates that the various elements of the bovine interplacentomal wall of the gravid uterine horn, as we hypothesized, are subjected to profound histomorphological changes during pregnancy. These alterations, which were already visible at the light microscopic level (Figure 1), were quantified by measurements using a calibrated computerized microscope measuring device and were found to be significant for many of the parameters evaluated (see Table 1). For interpretation of absolute values, it has to be considered that potential shrinkage artefacts due to histological preparation cannot be excluded. However, since all groups were subjected to exactly the same methodology, it is likely that relative data as well as group comparisons and thus developmental changes during pregnancy were unaffected.

The important role of the uterine SE is underlined by its continuous presence as a lining throughout pregnancy. This is in contrast to an early report stating that the SE is completely lacking during early pregnancy in cattle [24]. As evidenced by its expression of genes for many proteins, such as uterine milk proteins (UTMP) [25], glucose transporter-1 [26,27], calbindin D9k [28,29], transient receptor potential channel type 6 [29], MMP-2 and 9 [21], SOLD1 [30], TIMP-2 [21], EMMPRIN, an inducer of MMPs [31], galectins 3, 4, and 9 [32], and COX-2 [33,34], SE is involved in numerous functions during different phases of pregnancy, such as histiotrophic nourishment of the fetus, materno-fetal glucose and calcium transfer, endometrial restructuring, immunomodulation in favor of the fetus, and oxytocin-induced prostaglandin F2alpha production at term. The expression of receptors for estrogens [15,35] growth hormone [36], various elements of the IGF system [37], prostaglandins E and F2alpha [33,34], oxytocin [38] and glucocorticoids [15,35], sheds a light on various hormones and other factors of maternal and fetal origin influencing SE function during pregnancy. The lack of progesterone receptors in the SE during pregnancy is overcome by progesterone-sensitive subepithelial stromal cells producing paracrine-acting factors, such as growth factors [37], which in turn regulate SE function *via* their receptors expressed on SE and thus may also initiate SE hypertrophy and hyperplasia. Thus, the continuous presence of the SE is required during the whole of gestation. The highly active protein synthesis machinery and the secretion of uterine proteins [25,39] are reflected in the increasing SE height during early pregnancy, reaching a plateau by the 2nd trimester and maintaining this height until term. This pattern is in contrast to an early histomorphological description indicating no differences in SE height during pregnancy [11] and also to the maternal crypt epithelium of the bovine placentome, which becomes flattened and discontinuous towards term, a process named “placental maturation” [40]. Desquamated apoptotic SE cells may also be engulfed by the chorion, and thus contribute to histiotrophe in a holocrine manner [15,41].

EG play important roles during the entire course of pregnancy. This is reflected by significant increases in the height of the EG cells and in the lumen and gland diameters found in IG and DG in this study, objectifying earlier non-quantitative histomorphological descriptions and measurements that were inadequately statistically analysed [11,13]. Interestingly, as detected for the first time in this study, the Vv of EG cells did not change during pregnancy and this supports the concept of their consistent physiological relevance during all stages of pregnancy. The increase in IGE/DGE diameter and height therefore should be achieved by a reduction in their length. Endometrial secretions, called uterine milk (UTM) or histiotrophe, play an important role especially during early pregnancy, as demonstrated in the uterine gland knock-out ewe (UGKO), in which normal hatched blastocysts fail to undergo further development during the peri-implantation period since osteopontin and GlyCAM-1 are absent in uterine secretions of UGKO ewes. These proteins are essential for implantation in the ewe because they serve as ligands for integrins or as factors that affect their affinity state at the maternal-conceptus interface (see *e.g.*, [3]). UTM proteins are important for blastocyst elongation [27,39] and contain immunosuppressive components protecting the embryo from attacks by the maternal immune system [42]. They are secreted mainly by EG cells [25,43,44] during the entire pregnancy [14]. The amount of secretions, however, decreases with ongoing gestation and is paralleled by *in vitro* endometrial oxygen consumption, which is higher than that observed in caruncular, cotyledonary and intercotyledonary chorioallantois tissues [6].

EG epithelial cells exhibit a decreasing immunohistochemical staining for progesterone receptors with ongoing pregnancy, together with low levels of estrogen receptor immunoreactivity, while glucocorticoid receptor staining increases with ongoing pregnancy in glandular openings [15]. Furthermore, EG are also supplied with growth hormone receptors [36], IGF-I receptor [37,45] and prostaglandin E and F2alpha receptors [33,34] and thus are regulated directly by progesterone plus the same hormones and factors as the SE. Proteins expressed by the SE such as uterine milk proteins (UTMP) [25], glucose transporter-1 [26,27], calbindin D9k [28,29], transient receptor potential channel type 6 [29], MMP-2 and 9 [21], TIMP-2 [21], galectins 3, 4, and 9 [32], COX-2 [33,34], which have all been detected immunohistochemically in EG epithelia, indicate that EG are, similar to the SE, also involved in histiotrophic nourishment of the fetus, materno-fetal calcium transfer, endometrial restructuration, immunomodulation, and oxytocin-induced prostaglandin F2alpha production at term.

As for the SE, hyperplasia seems to play a minor role in glandular increases in diameter since small numbers of mitoses could be detected in only a few specimens and apoptosis is a sporadic event. This is supported by earlier, non-quantitative histomorphological [11,13] and semi-quantitative Ki-67 immunohistochemical studies [15] and is also reflected by generally low numbers of mitotic cells in the endometrium detected by flow cytometry [10].

The formation of a subepithelial mesh of connective tissue and the tremendous increase in diameter of the IGE and DGE necessitates a massive remodelling of the endometrial connective tissue, which is reflected by the expression of MMP-2 and −9 [46]. MMP-2 was localized immunohistochemically within the subepithelial mesh and in periglandular regions, while MMP-9 and TIMP-2 were detected in the SE and EG cells [21]. The mesh increases in thickness during pregnancy months 1 to 5 and possibly constricts glandular openings, which therefore cannot increase in diameter, as do IG and DG. It was analysed immunohistochemically and found to be composed mainly of collagen type I and III fibres and therefore should increase the tensile strength of the uterine wall [16]. Stromal cells within this layer also contained smooth muscle actin and were surrounded by collagen type IV, implying transformation of these cells to myofibroblasts [21]. During pregnancy, endometrial stromal cells express receptors for progesterone and are thus the main cell type mediating progesterone action within the endometrium. Secretions of EG, which are supplied with minimal amounts of progesterone receptors, or even completely lacking them, are activated and sustained *via* a servomechanism including progesterone-sensitive periglandular stromal cells producing paracrine factors, such as growth factors [2,37,45]. Estrogens, glucocorticoids [15,35], PGs E and F_2α_ [33,34] and growth hormone [36] directly regulate endometrial stromal function *via* their receptors. In this study, endometrial stroma Vv decreased significantly with ongoing pregnancy (67.2 *vs.* 37.0%), reflecting the five-fold increase in EG lumen Vv (8.2 *vs.* 40.1%). Thus, the endometrial stroma distribution pattern profoundly changes during the first half of pregnancy.

The main changes in myometrial histomorphology, *i.e.*, significant increase in MSMC diameter and significant decrease in nucleus:cytoplasm ratio of MSMC, besides the absence of mitoses in MSMC, strongly suggest hypertrophy and not hyperplasia of these cells during pregnancy. This is in agreement with early microscopic descriptions, which were not verified by measurements [11,12], and a study using Ki-67 immunohistochemistry [15]. These results are, however, in contrast to the rat myometrium, which exhibits a proliferative phase lasting from gestational days 6 to 14 [47]. In sheep and rat, the increase in MSMC cytoplasm is reflected by increases in actin and in smooth muscle myosin heavy chain SM2 protein and other contraction-associated proteins [18-20,48]. Structural elements of the MSMC cytoskeleton parallel these events [49], which are completed by an increase in gap junction-forming connexin 43 protein immediately preterm [48]. This final step transforms the myometrium into an electrical syncytium capable of synchronized contractions, *i.e.*, exhibiting a parturient pattern of myometrial activity, and expulsion of the fetus [50,51]. Besides the increase in size of MSMC because of the cytoplasmic accumulation of cytoskeletal and contraction-associated proteins, the synthesis of these proteins and of ECM materials, of receptors for ovarian steroids and other hormones and the secretion of prostaglandins may to varying degrees also contribute to the enlargement of these cells [15,33,37,47,48,52-55].

There was a continuous increase in myometrial stroma Vv in the cows of this study. Collagen types I and III slightly increase with ongoing pregnancy [16]. It was shown in the rat that, besides elastin, collagen types I and III are continuously synthesized until the preterm fall in progesterone, when basement membrane proteins such as collagen type IV and laminin ß2 are synthesized together with fibronectin and integrins [17,47,49], thus coupling the contractile apparatus *via* cytoskeleton and basement membrane to the fibrillar collagens of the myometrial stroma, resulting in a system of great tensile strength and contractile force.

## Conclusions

The results of this study indicate that the structural elements of the interplacentomal uterine wall, assessed quantitatively and appropriately analysed statistically for the first time, exhibit changes during pregnancy characteristic of hypertrophy, reflecting the needs of the growing fetus and its expulsion at term. They also emphasize the role of the interplacentomal uterine wall, in particular its SE and EG.
